# Stochastic forecasting of variable small data as a basis for analyzing an early stage of a cyber epidemic

**DOI:** 10.1038/s41598-023-49007-2

**Published:** 2023-12-20

**Authors:** Viacheslav Kovtun, Krzysztof Grochla, Vyacheslav Kharchenko, Mohd Anul Haq, Andriy Semenov

**Affiliations:** 1 Present Address: Institute of Theoretical and Applied Informatics, Polish Academy of Sciences, Gliwice, Poland; 2 National Aerospace University KhAI, Kharkiv, Ukraine; 3 College of Computer and Information Sciences, Majmaah University, Al Majma’ah, Saudi Arabia; 4 Vinnytsia National Technical University, Vinnytsia, Ukraine

**Keywords:** Computer science, Information technology

## Abstract

Security Information and Event Management (SIEM) technologies play an important role in the architecture of modern cyber protection tools. One of the main scenarios for the use of SIEM is the detection of attacks on protected information infrastructure. Consorting that ISO 27001, NIST SP 800-61, and NIST SP 800-83 standards objectively do not keep up with the evolution of cyber threats, research aimed at forecasting the development of cyber epidemics is relevant. The article proposes a stochastic concept of describing variable small data on the Shannon entropy basis. The core of the concept is the description of small data by linear differential equations with stochastic characteristic parameters. The practical value of the proposed concept is embodied in the method of forecasting the development of a cyber epidemic at an early stage (in conditions of a lack of empirical information). In the context of the research object, the stochastic characteristic parameters of the model are the generation rate, the death rate, and the independent coefficient of variability of the measurement of the initial parameter of the research object. Analytical expressions for estimating the probability distribution densities of these characteristic parameters are proposed. It is assumed that these stochastic parameters of the model are imposed on the intervals, which allows for manipulation of the nature and type of the corresponding functions of the probability distribution densities. The task of finding optimal functions of the probability distribution densities of the characteristic parameters of the model with maximum entropy is formulated. The proposed method allows for generating sets of trajectories of values of characteristic parameters with optimal functions of the probability distribution densities. The example demonstrates both the flexibility and reliability of the proposed concept and method in comparison with the concepts of forecasting numerical series implemented in the base of Matlab functions.

## Introduction

The era of computer viruses lasted little more than 40 years^1–5^. One of the first viruses was developed for an Apple computer. It happened in 1981, and the name of the "progenitor" was Elk Cloner. This virus was not so much harmless as annoying: with each download, the user of the infected computer saw a funny (in the opinion of the cyberbully) poem on the screen, after which the computer worked in normal mode. The first widespread virus for computers running the MS-DOS operating system appeared in 1986 and was called Brain. However, the developers of this virus, Pakistani brothers Farooq Alvi, did not want to harm people: they wrote Brain to protect the medical program they created from unlicensed copying. Computer viruses have come a long way since their inception, and today's malicious programs are much more subtle than their counterparts from the 80s and 90s and are much more difficult to detect. In this regard, computer viruses are very similar to their biological "brothers". Today, users may not notice for years that a program is running on their gadget, which either silently collects information or forces the user's device to perform certain actions, or masks the actions of other, much more dangerous programs. Each type of pest has its name and is intended for attackers to achieve various selfish goals^6–10^.

One of the earliest computer virus epidemics happened as far back as 1988, when the "big worm" or the Morris worm, named after its author, Robert Morris, spread over the Arpanet network in the United States. The worm, picking up passwords, filled the computers of network users with its copies and thus managed to infect more than 6 k computers, causing about 100 million dollars in damages—a colossal amount for those times. Since then, cyber epidemics have occurred repeatedly. For example, a real nightmare for bankers was the Carbanak virus, which in 2014 caused losses to American, Swiss, Dutch, Japanese and Ukrainian banks totalling more than 1 billion dollars. Carbanak moved slowly but surely, first gathering data from rank-and-file bank employees it reached through email attachments, then working its way up the ranks and withdrawing large sums of money. It could take from 2 to 4 months from the penetration into the bank system to a successful transaction. Here is a more recent example. More than 500 k computer users were affected by the WannaCry blocker, which appeared in May 2017. This extortionist, very common in Ukraine and India, encrypted information on the computer and demanded money for unlocking. Penetration into the system occurs through open TCP ports. The worm itself did not choose its victims based on certain characteristics, so it paralyzed the work of both ordinary users and various institutions. Comprehensive reporting of cyber outbreaks is worthy of a separate review. Besides, every day, the world-renowned Institute AV-TEST registers almost half a million new malicious and potentially unwanted programs: https://www.av-test.org/en/statistics/malware/ The provided information is enough for us to state that cyber epidemics are a formidable reality that needs the attention of scientists.

However, research is already underway. Unification is one of the pillars of technological society. Not surprisingly, the scientific community uses the same arsenal to describe both biological virus epidemics and computer virus epidemics. The *SIR* model^1,3,11–14^, which is not mentioned in almost any article on epidemiology, was created almost a century ago. It is simple and elegant, but it is much criticized and much praised, like any entity that is used only in certain conditions and has its limitations that you need to be aware of when using it. This model assumes the division of the population that is prone to an epidemic into three groups: *S* (susceptible that does not have immunity), *I* (infected, contagious) and *R* (recovered, not contagious, immune). However, the nomenclature is not limited to the three mentioned groups. For example, in the *SEIR* model^15^, group *E* (exposed-infected, not contagious) was added to the three mentioned groups. All these models are instances of the family of compartmental epidemiological models^16–20^. The above-mentioned division of the population into groups or compartments is precisely what determines this family name. The complexity of compartmental models is not limited to three or four groups. Such models can take into account different scenarios: introduction of quarantine measures (*SIQR*, added group *Q*—quarantine), loss of immunity (*SIRS*, transition with some probability from group *R* back to group *S*), risk groups according to susceptibility (several groups *S*, each with its probability of infection), different options for the course of infection (several groups *I*, each with its probability of hitting susceptible members of the population), etc. The only limitation here is the researchers' imagination. Complex schemes of compartmental models are usually presented in the form of transition graphs^21–23^. The implementation of compartmental models consists of the formalization of transition equations and the subsequent calculation of changes in each compartment during a certain finite period. If we assume that the number of susceptible to infection and infected is a random variable, then the model becomes stochastic. Typically, such a model is created to answer the question: if the number of uninfected members of the population was equal to $x$ in that week, how will the value of this parameter change this week? What is the probability of avoiding infection $u$? If we take into account that $u$ depends on the number of infected members of the population, then we get the Reed Frost model^24–26^. If it is assumed that the probability of infection does not depend on the number of infected, then we get the Greenwood model^27–30^. These are more flexible, non-continuous models. They are also called branching models^24–30^. Stochastic models became widespread in the 1970s, are constantly evolving and now generally agree with the results of observations of the course of various biological infections.

However, in the case of describing computer virus epidemics or cyber epidemics, the acquired "biological" experience is not inherited. Experience modelling a flu epidemic will not help when simulating the WannaCry cyber epidemic. The main reason for this is the fundamental difference in the spaces in which members of fundamentally different populations live and interact. In this context, the mathematical apparatus of time series analysis is suitable for describing the development of cyber epidemics^34–38^. However, the disadvantage of this approach is the strength of compartmental models—time series analysis models do not take into account the specifics of the development of the cyber epidemic.

Machine learning is a powerful tool for determining the relationship between input and output data for processes that are difficult to analyze analytically. The use of heuristic approaches for the early detection of epidemiological risks in some cases allows to improve the quality of forecasting. Among the examples of machine learning models in the context of the subject of the article, we note dynamic Bayesian networks^31–33^. This architecture is based on a directed graph, the vertices of which correspond to the parameters of the researched process, and the edges to probabilistic dependencies between these independent parameters are defined in the form of certain distribution laws. After training on both a large and a small amount of initial data, Bayesian networks make it possible to estimate the probability of the occurrence of some event in the context of the studied sequence of phenomena. To predict the development of biological epidemics, a simple form of the hidden Markov model is used, which is based on the idea of comparing each random variable $Y\left( t \right)$ (for example, the number of computer network nodes with declared atypical behaviour) with a random unmeasured variable $S\left( t \right)$ (for example, the total number of computer network nodes with both recorded virus infection and heuristically determined signs of the latter), which characterizes the conditional distribution $Y\left( t \right)$. Thus, the value $Y\left( t \right)$ depends only on the value of the hidden variable $S\left( t \right)$ at time $t$, and the sequence $S\left( t \right)$ has the Markov property, that is, the value $S\left( t \right)$ depends only on $S\left( {t - 1} \right)$. The need to fulfil these and several other mandatory conditions limit the applied change of this approach in the context of the phenomenon to which this article is devoted.

Artificial neural networks^32–34^ are directed weighted graphs, the vertices of which model the functioning of biological neurons or their functionally oriented populations. The training of such models consists of calculating the coefficients of connections between vertices, which determine the strength of the input signals and is performed based on empirical data: infection statistics based on the values of the factors that determine it. For the correct training of such neural networks, a large amount of representative data is needed, which simply cannot be in the case of a new cyber epidemic.

Any time series of morbidity can be considered as a random process consisting of a signal reflecting the real epidemic situation and high-frequency noise. Noise filtering allows us to refine the prediction and can be performed both during the pre-processing of the raw data and directly in the body of the prediction algorithm. One such approach is wavelet decomposition^35,36^, in which a short time series is represented by wavelet functions. This approach is usually used in conjunction with other models. One such model is exponential smoothing, which is a special case of the weighted moving average, and the incidence value $y\left( t \right)$ at time t is described by the weighted sum of the last observations: $by\left( t \right) + \left( {1 - b} \right)y\left( {t - 1} \right)$, where $b \in \left( {0,1} \right)$ is a smoothing factor that provides weight reduction as the data ages, which can be considered as a reflection of the natural learning process. This method of model creation is suitable for series whose behaviour shows a clear trend or seasonality. These conditions for cyber epidemics are fulfilled only in the abstract.

T. Schelling in 1971 and M. Mitchell in 1993 proposed the theory of cellular automata to model the local characteristics of susceptible populations together with stochastic parameters that reflect the probabilistic nature of the development of a biological epidemic. Cellular automata are considered as a set of square cells united in a rectangular grid, each cell of which takes a state from a finite set. Grid nodes model entities-individuals, each of which has a fixed position in space. This approach allows us to focus on the contribution of the human factor to the process of the development of a cyber epidemic. The description of the process of computer network node infection in terms of probabilistic cellular automata and ordinary differential equations has a perspective and will be investigated by the authors in the following works.

Patrolla in 2004 proposed an agent-oriented model^37,38^, which expands the capabilities of cellular automata in the context of tracking the spread of infection, taking into account mutual contacts between individuals united in a certain social group. Such a model is embodied in the scheme of possible contacts as a dynamic or static graph, the vertices of which correspond to objects with a finite, but sufficiently detailed, set of individual properties inherent to individuals or their classes. This is a potentially promising approach in the context of the subject of this article, but it requires the presence of very specific a priori information for its implementation. This fact does not allow the mathematical apparatus of agent-oriented models to claim universality in the contest of the thematics of this research.

Thus, there is no ready universal solution for describing the development of the cyber epidemic. This fact opens up great prospects for scientific research.

Considering the merits and limitations of the aforementioned approaches, we shall now outline the essential characteristics or attributes that scientific research should possess.

The *object* of study is the process of the development of a cyber epidemic at an early stage.

The *subject* of study encompasses probability theory and mathematical statistics, information theory, the theory of experiment planning, mathematical programming methods, and numerical methods.

Dear reader, for a more complete understanding of the mathematics-rich material in “Models and methods” section, we recommend that you first read the article^40^, which reveals the theoretical background of the applied research to which this article is devoted.

The *aim* of the study is to formalize the process of finding optimal functions of probability distribution densities of stochastic characteristic parameters of the variable small data description model with maximum entropy in the context of the problem of forecasting the development of a cyber epidemic at an early stage.

The *objectives* of the study are:

- to formalize the concept of calculating variable entropy estimation for functions derived from probability distribution densities of characteristic parameters within a stochastic model. This model is used to describe variable small data, which is represented by interval normalized probabilities.;
- to formalize the process of forecasting the development of cyber epidemics in terms of the stochastic-entropy concept of the description of variable small data;
- to justify the adequacy of the proposed mathematical apparatus and demonstrate its functionality with an example.

The *main contribution*. The article proposes a stochastic concept of describing variable small data on the Shannon entropy basis. The core of the concept is the description of small data by linear differential equations with stochastic characteristic parameters. The practical value of the proposed concept is embodied in the method of forecasting the development of a cyber epidemic at an early stage (in conditions of a lack of empirical information). In the context of the research object, the stochastic characteristic parameters of the model are the generation rate, the death rate, and the independent coefficient of variability of the measurement of the initial parameter of the research object. Analytical expressions for estimating the probability distribution densities of these characteristic parameters are proposed. It is assumed that these stochastic parameters of the model are imposed on the intervals, which allows for manipulation of the nature and type of the corresponding functions of the probability distribution densities. The task of finding optimal functions of the probability distribution densities of the characteristic parameters of the model with maximum entropy is formulated. The proposed method allows for generating sets of trajectories of values of characteristic parameters with optimal functions of the probability distribution densities.

The *highlights* of the study are:

- the instances of the class of parameterized stochastic models for the description of variable small data,
- the methods of estimating the functions of the probability distribution densities of their parameters, represented by interval probabilities,
- an approach to generating trajectories of random vectors of initial parameters of the model and their statistical processing by the Monte Carlo method to determine numerical characteristics with maximum entropy,
- a method of forecasting the development of a cyber epidemic in terms of the stochastic-entropy concept of describing variable small data.

## Models and methods

### Setting of the research

Let's examine an object with input parameters $x\left( t \right) = \left\{ {x_{i} \left( t \right)} \right\}$, output parameters $y\left( t \right) = \left\{ {y_{i} \left( t \right)} \right\}$ and parameters $\varepsilon \left( t \right) = \left\{ {\varepsilon_{i} \left( t \right)} \right\}$ that characterize the variability of measurements of output parameters, $i = \overline{1,n}$, $t \in T_{p} = \left[ {t_{0} ,T} \right]$, $t_{0} < T$. We describe the object with a dynamic model with input parameters $x\left( t \right) = \left\{ {x_{i} \left( t \right)} \right\}_{{}}^{{}}$ and output parameters $f\left( t \right) = \left\{ {f_{i} \left( t \right)} \right\}$, $i = \overline{1,n}$, $t \in T$. We define the censored observation interval for the object and the model as $T_{tr} = \left[ {T_{ - } ,t_{e} } \right) \cup \left[ {t_{e} ,t_{0} } \right)$, where $T_{e} = \left[ {T_{ - } ,t_{e} } \right)$ is the training data collection interval, and $T_{t} = \left[ {t_{e} ,t_{0} } \right)$ is the test data collection interval, $T_{e} < t_{e} < t_{0}$. The parameters of the mentioned dynamic model are stochastic values. Characteristic features of this model will be the probability distribution densities of these stochastic parameters.

The optimal evaluation of the desired probability distribution densities can be carried out based on data collected at the interval $T_{e}$. We will use the data collected at the interval $T_{t}$ to test the model. On the interval $T_{p}$, we will forecast the object-process using the model $f\left( t \right) \to y\left( t \right)$. Let us formalize the connection between parameters $x\left( t \right)$ and $f\left( t \right)$ in the form of a system of linear differential equations:

1 $$\frac{df\left( t \right)}{{dt}} = C^{\left( f \right)} f\left( t \right) + C^{\left( x \right)} x\left( t \right),$$

at $$f\left( {T_{ - } } \right) = f_{0}$$, where $$f \in R^{n}$$, $$x \in R^{m}$$, $$n \ge m$$; $$C^{\left( f \right)} = \left[ {c_{ij}^{\left( f \right)} } \right]$$, $$i,j = \overline{1,n}$$; $$C^{\left( x \right)} = \left[ {c_{ik}^{\left( x \right)} } \right]$$, $$i = \overline{1,n}$$, $$k = \overline{1,m}$$.

The resulting output of the model (1) will be described as

2 $$o\left( t \right) = f\left( t \right) + \varepsilon \left( t \right).$$

Let's formulate the following requirements:

*R1*. The matrix $C^{\left( f \right)}$ is formed by stochastic elements of the interval type

3 $$C = \left[ {C:C_{ - }^{\left( f \right)} \le C^{\left( f \right)} \le C_{ + }^{\left( f \right)} } \right],$$

where $C_{ - }^{\left( f \right)}$, $C_{ + }^{\left( f \right)}$ are the applied matrices, the elements of which can be both stochastic quantities and linear combinations of a finite number of stochastic quantities;

*R2*. The elements of the matrix $C^{\left( x \right)}$ are known and fixed;

*R3*. The probability distribution density $P\left( C \right)$ exists, $\forall C^{\left( f \right)} \in C$;

*R4*. Vectors $\varepsilon \left( t \right)$, $t \in T_{e}$, are formed by independent components of the interval type:

4 $${\rm E} = \left[ {\varepsilon_{ - } \le \varepsilon \le \varepsilon_{ + } } \right].$$

If the conditions *R1*–*R4* are fulfilled, then model (1) allows obtaining a set of trajectories (2) for the stochastic parameter $f\left( t \right)$, $t \in \left\langle {T_{e} ,T_{t} ,T_{p} } \right\rangle$.

Let us rewrite expression (1) taking into account the existence of the fundamental matrix of solutions^39^:

5 $$V\left( {C^{\left( f \right)} \left| {t - \tau } \right.} \right) = \exp \left( {C^{\left( f \right)} \left( {t - \tau } \right)} \right).$$

Based on expression (5), we write:

6 $$f\left( t \right) = V\left( {C^{\left( f \right)} \left| {t - T_{ - } } \right.} \right)f_{0} + \int\limits_{{T_{ - } }}^{t} {V\left( {C^{\left( f \right)} \left| {t - \tau } \right.} \right)C^{\left( x \right)} x\left( \tau \right)d\tau } .$$

If the measurement of "input–output" entities is carried out at discrete moments with a step $\Delta$, then at the interval $T_e$ the expression (6) will take the form

7 $$f\left( {T_{ - } + i\Delta } \right) = V\left( {C^{\left( f \right)} \left| {i\Delta } \right.} \right)f_{0} + \int\limits_{{T_{ - } }}^{{T_{ - } + i\Delta }} {V\left( {C^{\left( f \right)} \left| {T_{ - } + i\Delta - \tau } \right.} \right)C^{\left( x \right)} x\left( \tau \right)d\tau } ,$$

where $$i \in \overline{{1,N_{e} }}$$, $$N_{e} = \left\lfloor {{{\left( {t_{e} - T_{ - } } \right)} \mathord{\left/ {\vphantom {{\left( {t_{e} - T_{ - } } \right)} \Delta }} \right. \kern-0pt} \Delta }} \right\rfloor$$.

Let's rewrite expression (2) taking into account expression (7):

8 $$o\left( {T_{ - } + i\Delta } \right) = f\left( {T_{ - } + i\Delta } \right) + \varepsilon \left( {T_{ - } + i\Delta } \right),$$

where $i \in \left[ {0,N_{e} } \right]$. For compactness, we denote the block vector $\varepsilon \left( {T_{ - } + i\Delta } \right)$ with dimension $n \times \left( {N_{e} + 1} \right)$ mentioned in expression (8) as $\varepsilon^{\left( e \right)} = \left\{ {\varepsilon^{\left( k \right)} } \right\}$, $k = \overline{{0,N_{e} }}$.

Taking into account the a priori independence of both vectors $\varepsilon^{\left( e \right)}$ and their elements, we define the compatible probability distribution density as $Q\left( {\varepsilon^{\left( e \right)} } \right) = \prod\nolimits_{i = 0}^{{N_{e} }} {Q_{i} \left( {\varepsilon^{\left( i \right)} } \right)} = \prod\nolimits_{i = 0}^{{N_{e} }} {\prod\nolimits_{j = 1}^{n} {q_{ij} \left( {\varepsilon_{j}^{\left( i \right)} } \right)} }$ with the definition domain of ${\rm E}^{\left( e\right)} = {\rm E}^{{N_{e} + 1}}$.

Therefore, with a defined matrix *C*, which is characterized by the probability distribution density $P\left( C \right)$, and the vector of the variability of the measurements of the output parameters $\varepsilon^{\left( e \right)}$, which is characterized by the compatible probability distribution density $Q\left( {\varepsilon^{\left( e \right)} } \right)$, expression (8) is the basis for obtaining a set of the desired stochastic trajectories $o\left( {T_{ - } + i\Delta } \right)$, $i \in \left[ {1,N_{e} } \right]$.

### Stochastic concept of the description of variable small data in Shannon entropy basis

We formalize the estimation of the optimal probability distribution densities $P^{ * } \left( C \right)$ and $Q^{ * } \left( {\varepsilon^{\left( e \right)} } \right)$ in terms of the stochastic model for variable small data evaluation in the Shannon entropy basis, which the authors presented in^40^. We define the objective function of the optimization problem as

9 $$H\left( {P\left( C \right),Q\left( {\varepsilon^{\left( e \right)} } \right)} \right) = - \int\limits_{C} {P\left( C \right)\ln P\left( C \right)dC} - \int\limits_{{{\rm E}^{\left(e \right)} }} {Q\left( {\varepsilon^{\left( e \right)} } \right)\ln Q\left( {\varepsilon^{\left( e \right)} } \right)d\varepsilon^{\left( e \right)} } \to \max .$$

We specify the system of limitations of such an optimization problem. The first limitation is obvious and focused on the normalization of the investigated probability distribution densities:

10 $$\int\limits_{C} {P\left( C \right)dC = 0.5} ,\quad \int\limits_{{{\rm E}^{\left( e\right)} }} {Q\left( {\varepsilon^{\left( e \right)} } \right)d\varepsilon^{\left( e \right)} } = 1.$$

The second limitation is focused on ensuring the adequacy of the model to the studied process and is aimed at maintaining a balance between the output parameter of the object $y\left( t \right)$ and the output parameter of the model $o\left( t \right)$. Let's formulate this balance equation for the discrete form of representation of the corresponding characteristic parameters, that is, for $y\left( {T_{ - } + i\Delta } \right) = y^{\left( i \right)}$ and $o\left( {T_{ - } + i\Delta } \right) = o^{\left( i \right)}$, $i \in \left[ {0,N_{e} } \right]$:

11 $$y^{\left( i \right)} = M\left\{ {o^{\left( i \right)} } \right\} = \int\limits_{C} {w^{\left( i \right)} P\left( C \right)dC} + \int\limits_{{{\rm E}^{\left( e \right)} }} {\varepsilon^{\left( i \right)} Q\left( {\varepsilon^{\left( e \right)} } \right)d\varepsilon^{\left( e \right)} } ,$$

where $M\left\{ {o^{\left( i \right)} } \right\}$ is the first moment of the parameter $o^{\left( i \right)}$ (look at the expression (5) in the author's work^40^), and the parameter $w^{\left( i \right)}$ is determined by the expression

12 $$\begin{aligned} w^{\left( i \right)} \left( C \right) & = V\left( {C\left| {i\Delta } \right.} \right)f_{0} \\ & \quad + \int\limits_{{T_{ - } }}^{{T_{ - } + i\Delta }} {V\left( {C + i\Delta - \tau } \right)C^{\left( x \right)} x\left( \tau \right)d\tau } , \\ \end{aligned}$$

and $\int_{C} {w^{\left( i \right)} P\left( C \right)dC} \le 0.5$, $\int_{{{\text{E}}^{\left( e \right)} }} {\varepsilon^{\left( i \right)} Q\left( {\varepsilon^{\left( e \right)} } \right)d\varepsilon^{\left( e \right)} } \le 0.5$ and by manipulating the values of $w^{\left( i \right)}$, $\varepsilon^{\left( i \right)}$, respectively.

The balance Eq. (11) is formulated in the context of the independence of the elements of the vector $\varepsilon^{\left(e \right)}$.

The optimization problem with the objective function (9) and limitations (10), (11) can be classified as a global optimization problem^41^. The theory of global optimization summarizes a grand and constantly expanding pleiad of solution methods, which can be most generally segmented into three classes. The methods of the first class are focused on the configuration of the objective function and the set of admissible solutions. A characteristic representative of this class is the concept of *DC* minimization, in which the objective function and the limitations functions are represented by the differences between two convex functions. The methods of the second class investigate simple admissible sets and objective functions with a known Lipshitz constant. We will especially note the concept of reducing a $n$-dimensional problem to a *1*-dimensional one using Peano curves^41^. The third class of methods is based on the Monte Carlo method with various pseudo-intelligent heuristics^41,42^. In this class of methods, it is necessary to solve the problem of generating uniformly distributed stochastic vectors within the domain of the search space. For this, both numerous modifications of the Hit-and-Run concept^41,42^, as well as concepts based on Markov chains^43^ and concepts based on Kullback–Leibner entropy^32,41,42^ are used. Further analytical constructions will be formulated based on the methods of the third class.

The optimization problem with the objective function (9) and limitations (10), (11) belongs to the Lyapunov type because the functional-objective function and the limitations are integral. Let us analytically express the solution to this problem in terms of the concept of Monte Carlo for global optimization. We will get:

13 $$\begin{aligned} P^{ * } \left( C \right) = & {{\exp \left( { - \sum\limits_{i = 0}^{{N_{e} }} {\left( {\beta^{\left( i \right)} \cdot w^{\left( i \right)} \left( C \right)} \right)} } \right)} \mathord{\left/ {\vphantom {{\exp \left( { - \sum\limits_{i = 0}^{{N_{e} }} {\left( {\beta^{\left( i \right)} \cdot w^{\left( i \right)} \left( C \right)} \right)} } \right)} {{\mathbb{R}}\left( \beta \right)}}} \right. \kern-0pt} {{\mathbb{R}}\left( \beta \right)}}, \\ Q^{ * } \left( {\varepsilon^{\left( e \right)} } \right) = & {{\exp \left( { - \sum\limits_{i = 0}^{{N_{e} }} {\left( {\beta^{\left( i \right)} \cdot \varepsilon^{\left( i \right)} } \right)} } \right)} \mathord{\left/ {\vphantom {{\exp \left( { - \sum\limits_{i = 0}^{{N_{e} }} {\left( {\beta^{\left( i \right)} \cdot \varepsilon^{\left( i \right)} } \right)} } \right)} {{\mathbb{Q}}\left( \beta \right)}}} \right. \kern-0pt} {{\mathbb{Q}}\left( \beta \right)}}, \\ \end{aligned}$$

where $\beta = \left\{ {\beta^{\left(i \right)} } \right\}$ is the solution vector, $i = \overline{{0,N_{e} }}$, the sign "$\cdot$" represents the scalar product, and the functions ${\mathbb{R}}\left( \beta \right)$ and ${\mathbb{Q}}\left( \beta \right)$ are defined as

$${\mathbb{R}}\left( \beta \right) = \int\limits_{C} {\exp \left( { - \sum\limits_{i = 0}^{{N_{e} }} {\beta^{\left( i \right)} \cdot w^{\left( i \right)} \left( C \right)} } \right)dC} ,$$

$${\mathbb{Q}}\left( {\varepsilon^{\left( e \right)} } \right) = \int\limits_{\rm E} {\exp \left( { - \sum\limits_{i = 0}^{{N_{e} }} {\beta^{\left( i \right)} \cdot \varepsilon^{\left( i \right)} } } \right)d{\rm E}} ,$$

respectively.

By substituting expression (13) into expression (12), we express the vectors of Lagrange multipliers:

14 $$W\left( \beta \right) = \int\limits_{C} {w^{\left( i \right)} \left( C \right)\frac{{\exp \left( {\sum\limits_{i = 0}^{{N_{e} }} {\beta^{\left( i \right)} \cdot w^{\left( i \right)} \left( C \right)} } \right)}}{{{\mathbb{R}}\left( \beta \right)}}dC} + \int\limits_{\rm E} {\varepsilon^{\left( i \right)} \frac{{\exp \left( {\sum\limits_{i = 0}^{{N_{e} }} {\beta^{\left( i \right)} \cdot \varepsilon^{\left( i \right)} } } \right)}}{{{\mathbb{Q}}\left( \beta \right)}}d{\rm E}} - y^{\left( i \right)} = 0.$$

The optimal solution $\beta^{ * } = \left\{ {\beta^{ * \left( i\right)} } \right\}$, $i = \overline{0,N}_{e}$, of the system of Eq. (14) coincides with the global extremum of the discrepancy function $J\left( {\beta^{ * } } \right)$:

15 $$J\left( {\beta^{ * } } \right) = \left\| {W\left( {\beta^{ * } } \right)} \right\|.$$

The achievement of $\beta^{ * }$ marks the completion of training of the model (7) with the stochastic composite parameters $C$, $\varepsilon^{\left( e\right)}$ and the corresponding functions of probability distribution densities $P^{ * } \left( C \right)$ and $Q\left( {\varepsilon^{\left( e \right)} } \right)$, which are determined by the expressions (13). Parallelepiped-like regions of admissible values of the parameters $C$ and $\varepsilon^{\left( e\right)}$ are defined by expressions (3) and (4), respectively.

We will focus on the application of the trained model of description of variable small data in the Shannon entropy basis for forecasting. Forecasting based on the trained model (7) consists in generating stochastic matrices of parameters $C$ and $\varepsilon$ with functions of the probability distribution densities (13) for the interval $T_{p}$. Let's formalize this process. We move from the matrix to the vector form of the description of the characteristic parameter $C$. To do this, we will make a serial connection of the rows of the matrix, obtaining a vector $\alpha$ of the length $m = n^{2}$ of independent stochastic elements. The domain for the elements of the stochastic vector $\alpha$ will be defined by the $m$-dimensional parallelepiped ${\rm A} = \left[ {\alpha_{ - } \le \alpha \le \alpha_{ + } } \right]$, where the vectors $\alpha_{ - }$ and $\alpha_{ + }$ are the result of the matrix-to-vector transformation of the described above matrices $C_{ - }^{\left( f \right)}$ and $C_{ + }^{\left( f \right)}$, respectively. Let us introduce the vectors $q$ that belong to the positive unit cube ${\text{Q}}$: ${\text{Q}} = \left\{ {q:0 \le q \le 1} \right\}$. We connect the vectors $\alpha$ and $q$ by an analytic relation of the form $\alpha = q\left( {\alpha_{ + } - \alpha_{ - } } \right) + \alpha_{ - }$.

Based on the above, the optimal probability distribution density $P^{ * } \left( C \right)$ undergoes a sequence of transformations of the form

16 $$P^{ * } \left( C \right) \to P \left( \alpha \right) \to {\rm P}\left( q \right).$$

To generate stochastic vectors $q \in {\text{Q}}$ with probability distribution density ${\text{P}}\left( q \right)$, it is proposed to use the Acceptance-–ejection method^42^. This choice is justified by the fact that we assume the rational sufficiency of the procedures for measuring the characteristic parameters of the object at intervals $T_{e}$, $T_{t}$.

### Forecasting the development of the cyber epidemic in terms of the stochastic-entropy concept of the description of variable small data

Let's take a high-availability cluster^44,45^ as an environment for the start and development of a cyber epidemic. Consider the cluster as a closed system. Let's introduce the parameter $E\left( t \right)$, which characterizes the number of infected cluster nodes at a time $t$. The change in the number of infected nodes will be characterized by the variable $v\left( t \right) = {{dE\left( t \right)} \mathord{\left/ {\vphantom {{dE\left( t \right)} {dt}}} \right. \kern-0pt} {dt}}$. The dynamics of the change in the value of the parameter $v\left( t \right)$ are ensured by the combined influence of the streams of generation and death.

The generation flow is characterized by the parameter $B$ (the number of infected cluster nodes per unit of time). Symmetrically, the flow of death is characterized by the parameter $M$ (the number of infected nodes of the cluster that went into a neutral state as a result of the activity of individual defence mechanisms that coped with the cyber infection (hereinafter "disinfected") per unit of time). We emphasize the fact that we are focusing on the early stage of the spread of a new cyber infection when a unified mechanism for its neutralization has not yet been created. We will assume that both of these flows depend linearly on the total number of nodes in the cluster. Let's move on to the relative time dimension of real-time $t$ (this is convenient because information processes in modern cyber-physical systems of high integration are relatively fast):

17 $$\tau = {t \mathord{\left/ {\vphantom {t \Delta }} \right. \kern-0pt} \Delta }.$$

In the time–space defined by expression (17), the development of the cyber epidemic will be determined by a first-order differential equation of the form

18 $$\frac{dE\left( \tau \right)}{{d\tau }} = E\Delta \left( {b - m} \right),E_{0} = E\left( {\frac{{T_{ - } }}{\Delta }} \right)$$

where $b$ is the relative generation rate (the number of newly infected nodes per time quantum, relative to the total number of nodes), $m$ is the relative death rate (the number of disinfected nodes per time quantum, relative to the total number of nodes). In current differential models of the development of the cyber epidemic, those coefficients are considered constant at certain time intervals. We argue that it is more realistic to define these parameters as interval ones: $I_{b} = \left[ {b_{ - } ,b_{ + } } \right]$, $I_{m} = \left[ {m_{ - } ,m_{ + } } \right]$. This approach allows taking into account the a priori uncertainty inherent in these characteristic parameters. This uncertainty prompts us to interpret the entities $b$ and $m$ as stochastic parameters that take on values in the intervals $I_{b}$ and $I_{m}$ with the compatible function of the probability distribution density $P\left( {b,m} \right)$ and the additive interval variability of the measurements $\varepsilon = \left\{ {\varepsilon \left( i\right)} \right\}$, $i = \overline{0,\Delta }$, where $I$ the number of heuristic antivirus scanning procedures in the quantum of time $\Delta$. Those independent elements generalized by the stochastic vector $\varepsilon$ are characterized by the probability distribution density $Q\left( \varepsilon \right)$ which is defined on the set ${\text{E}} = \bigcup\nolimits_{j = 0}^{I} {{\text{E}}_{j} }$, ${\rm E}_{j} = \left[ {\varepsilon_{ - }^{\left( j \right)} ,\varepsilon_{ + }^{\left( j \right)} } \right]$:

$$Q\left( \varepsilon \right) = \prod\limits_{j = 0}^{I} {q_{j} \left( {\varepsilon \left[ {j\Delta } \right]} \right)}$$.

Let us analytically express the solution of Eq. (18) for $\tau \in T$, $T = \left[ {\tau_{ - } ,\tau_{0} } \right]$, $\tau_{ - } = {{_{ - } } \mathord{\left/ {\vphantom {{T_{ - } } \Delta }} \right. \kern-0pt} \Delta }$, $\tau_{0} = {{t_{0} } \mathord{\left/ {\vphantom {{t_{0} } \Delta }} \right. \kern-0pt} \Delta }$:

19 $$E\left( \tau \right) = E_{0} \exp \left( {\tau \left( {b - m} \right)} \right).$$

By analogy with expression (8), we interpret the change in the number of infected nodes $v\left( t \right)$ by taking into account expression (19):

20 $$v\left( {i\Delta } \right) = \Omega_{i} \left( {b,m\left| {E_{0} } \right.} \right) + \varepsilon \left( {i\Delta } \right),$$

where $$i \in \left[ {0,I} \right]$$, and

21 $$\Omega_{i} \left( {b,m\left| {E_{0} } \right.} \right) = E_{0} \exp \left( {i\Delta \left( {b - m} \right)} \right)\forall i \in \left[ {0,I} \right].$$

Note that it is a function (21) that causes the individuality of the model (20). For intervals $\left\langle {{\text{T}}_{e} ,{\text{T}}_{t} ,{\text{T}}_{p} } \right\rangle$, represented by corresponding vectors of measurement results of length $\left\langle {N_{e} + 1,N_{t} + 1,N_{p} + 1} \right\rangle$, model (20) will take the form.

22 $$v\left( {i\Delta } \right) = \Omega_{i} \left( {b,m\left| {E_{e} } \right.\left( 0 \right)} \right) + \varepsilon \left( {i\Delta } \right),\;i \in \left[ {0,N_{e} } \right],$$

23 $$v\left( {i\Delta } \right) = \Omega_{i} \left( {b,m\left| {E_{t} } \right.\left( 0 \right)} \right) + \varepsilon \left( {i\Delta } \right),\;i \in \left[ {0,N_{t} } \right],$$

24 $$v\left( {i\Delta } \right) = \Omega_{i} \left( {b,m\left| {E_{p} } \right.\left( 0 \right)} \right) + \varepsilon \left( {i\Delta } \right),\;i \in \left[ {0,N_{p} } \right],$$

where the generation rate $b$ and the death rate $m$ are stochastic parameters with the optimal compatible probability distribution density $P^{ * } \left( {b,m} \right)$, determined on the set $I_{b} \cup I_{m}$ by expression (13) at $i \in \left[ {0,N_{e} } \right]$ and by expression (16) at $i \in \left[ {0,N_{t} } \right],\left[ {0,N_{p} } \right]$; disturbance $\varepsilon \left( {i\Delta } \right)$ is a vector whose elements are stochastically independent quantities with probability distribution density $Q\left( \varepsilon \right)$, $i = \overline{0,I\Delta }$; parameters $E_{t} \left( 0 \right)$, $E_{p} \left( 0 \right)$ are constant coefficients that are assigned by experts.

Let's analyze the functions $P^{ * } \left( {b,m} \right)$, $Q^{ * } \left( \varepsilon \right)$ analytically, based on the material of the previous section.

The optimal compatible function of the probability distribution density for the generation coefficient $b$ and the death coefficient $m$ is expressed as

25 $$P^{ * } \left( {b,m} \right) = {{\prod\limits_{j = 0}^{{N_{e} }} {p_{j}^{ * } \left( {b,m\left| {\beta_{j} } \right.} \right)} } \mathord{\left/ {\vphantom {{\prod\limits_{j = 0}^{{N_{e} }} {p_{j}^{ * } \left( {b,m\left| {\beta_{j} } \right.} \right)} } {{\mathbb{R}}\left( {\beta \left| { \left( 0 \right)} \right.} \right)}}} \right. \kern-0pt} {{\mathbb{R}}\left( {\beta \left| E_e { \left( 0 \right)} \right.} \right)}},$$

where $$p_{j}^{ * } \left( {b,m\left| {\beta_{j} } \right.} \right) = \exp \left( { - \beta_{j} \Omega_{j} \left( {b,m\left| {E_{e} \left( 0 \right)} \right.} \right)} \right)$$ and

$$\begin{aligned} \mathbb{R}\left( {\beta |E_{e} (0)} \right) & = \int\limits_{{I_{b} \cup I_{m} }} {\coprod\limits_{{j = 0}}^{{N_{e} }} {\exp ( - \beta _{j} } } \\ &\quad \times \Omega _{j} (b,m|E_{e} (0)))dbdm. \\ \end{aligned}$$

The optimal function of the probability distribution density for the variability of measurements of the output characteristic parameters of the object $\varepsilon$ is expressed as

26 $$Q^{ * } \left( \varepsilon \right) = {{\prod\limits_{j = 0}^{{N_{e} }} {q_{j}^{ * } \left( {\varepsilon \left( {j\Delta } \right)\left| {\beta_{j} } \right.} \right)} } \mathord{\left/ {\vphantom {{\prod\limits_{j = 0}^{{N_{e} }} {q_{j}^{ * } \left( {\varepsilon \left( {j\Delta } \right)\left| {\beta_{j} } \right.} \right)} } {{\mathbb{Q}}\left( \beta \right)}}} \right. \kern-0pt} {{\mathbb{Q}}\left( \beta \right)}},$$

where $$q_{j}^{ * } \left( {\varepsilon \left( {j\Delta } \right)\left| {\beta_{j} } \right.} \right) = \exp \left( { - \beta_{j} \varepsilon \left( {j\Delta } \right)} \right)$$ and

$${\mathbb{Q}}\left( \beta \right) = \prod\limits_{j = 0}^{{N_{e} }} {\int\limits_{{\left( {\varepsilon_{ - } } \right)_{j} }}^{{\left( {\varepsilon_{j} } \right)_{ + } }} {\exp \left( { - \beta_{j} \varepsilon \left( {j\Delta } \right)} \right)d\varepsilon \left( {j\Delta } \right)} } = \prod\limits_{j = 0}^{{N_{e} }} {{{\left( {\exp \left( { - \beta_{j} \left( {\varepsilon_{ - } } \right)_{j} } \right) - \exp \left( { - \beta_{j} \left( {\varepsilon_{ + } } \right)_{j} } \right)} \right)} \mathord{\left/ {\vphantom {{\left( {\exp \left( { - \beta_{j} \left( {\varepsilon_{ - } } \right)_{j} } \right) - \exp \left( { - \beta_{j} \left( {\varepsilon_{ + } } \right)_{j} } \right)} \right)} {\beta_{j} }}} \right. \kern-0pt} {\beta_{j} }}} .$$

To determine the Lagrange multipliers, we express the balance Eq. (14) in terms of expressions (25), (26) for $i \in \left[ {0,N_{e} } \right]$:

27 $$\frac{{{\mathbb{N}}_{i} \left( {\beta \left| {E_{e} \left( 0 \right)} \right.} \right)}}{{{\mathbb{R}}\left( {\beta \left| {E_{e} \left( 0 \right)} \right.} \right)}} + \frac{1}{{{\mathbb{Q}}\left( \varepsilon \right)}} \times \int\limits_{\rm E} {\varepsilon \left( {i\Delta } \right)\prod\limits_{j = 0}^{{N_{e} }} {\exp \left( { - \beta_{j} \varepsilon \left( {j\Delta } \right)} \right)} d\varepsilon \left( {j\Delta } \right)} - E_{e} \left( {i\Delta } \right) = 0,$$

where

$${\mathbb{N}}_{i} \left( {\beta \left| {E_{e} \left( 0 \right)} \right.} \right) = \int\limits_{{I_{b} \cup I_{m} }} {\Omega_{i} \left( {b,m\left| {E_{e} \left( 0 \right)} \right.} \right)} \prod\limits_{j = 0}^{{N_{e} }} {\exp \left( { - \beta_{j} \Omega_{j} \left( {b,m\left| {E_{e} \left( 0 \right)} \right.} \right)} \right)} dbdm.$$

Let's open the second term from expression (27). We will get:

28 $$\begin{aligned} L_{i} \left( {\beta_{i} } \right) & = \left( {\exp \left( { - \beta_{i} \left( {\varepsilon_{ - } } \right)_{i} } \right)\left( {\left( {\varepsilon_{ - } } \right)_{i} + \beta^{ - 1} } \right)} \right)\left( {\exp \left( { - \beta_{i} \left( {\varepsilon_{ - } } \right)_{i} } \right) - \exp \left( { - \beta_{i} \left( {\varepsilon_{ + } } \right)_{i} } \right)} \right)^{ - 1} \\ & \quad - \left( { - \exp \left( { - \beta_{i} \left( {\varepsilon_{ + } } \right)_{i} } \right)\left( {\left( {\varepsilon_{ + } } \right)_{i} + \alpha^{ - 1} } \right)} \right) \times \left( {\exp \left( { - \beta_{i} \left( {\varepsilon_{ - } } \right)_{i} } \right) - \exp \left( { - \beta_{i} \left( {\varepsilon_{ + } } \right)_{i} } \right)} \right)^{ - 1} . \\ \end{aligned}$$

Taking into account the expression (28), we present the balance Eq. (27) in a compact form $i \in \left[ {0,N_{e} } \right]$:

29 $$G_{i} \left( \beta \right) = \frac{{{\mathbb{N}}_{i} \left( {\beta \left| {E_{e} \left( 0 \right)} \right.} \right)}}{{{\mathbb{R}}\left( {\beta \left| {E_{e} \left( 0 \right)} \right.} \right)}} + L_{i} \left( {\beta_{i} } \right) - E_{e} \left( {i\Delta } \right) = 0.$$

We obtain the roots of Eqs. (29) $\forall i \in \left[ {0,N_{e} } \right]$ by analogy with expression (15) as a result of minimizing the discrepancy $J\left( \beta \right)$:

30 $$J\left( \beta \right) = \left\| {G\left( \beta \right)} \right\|_{{_{2} }} \to \min ,$$

where || || is interpreted as the Euclidean norm.

The dimensionality of the optimization problem with the objective function (30) is equal to $N_{e} + 1$. The actual complexity of the function (30) makes further analytical research of its properties impossible.

## Results

Experimental studies with the mathematical apparatus proposed in “Models and methods” will begin with the analysis of the focus group of "consumers". In the architecture of modern cyber protection tools, SIEM is undoubtedly such a "consumer"^41–43^. Classic SIEM is a log collector that collects events from such sources as DLP systems, firewalls, IPS, servers, workstations, routers, etc. and performs their analysis to detect information security incidents. The main scenarios of using SIEM include the detection of attacks in the early stages, automatic mapping of IT infrastructure, real-time monitoring of the state of IT infrastructure, detection and investigation of information security incidents, detection of new types of threats, optimization of the security monitoring model, etc. At the same time, it is important to understand that SIEM is not a means of protection as such. It is a nested logic and statistics-driven integrator tool for, sometimes, unrelated tools and functions, the purpose of which is to automate end-to-end information security processes. It is rational to implement SIEM if:—$\ge 1$ k computing devices are accepted in joint organizational activities;—basic means of information protection are implemented and functioning, for example, an antivirus system, UTM and/or IDS\IPS, DLP, Web proxy, etc.;—there is a need to reduce the intervention of the "human factor" in the processes of the information security service;—there is a need to ensure efficiency, reasonableness and integrity of the decision-making process in the field of information security;—it is necessary to ensure compliance of the protected cyberinfrastructure with ISO 27001, NIST SP 800-61 and NIST SP 800-83 standards.

**Figure 1:**
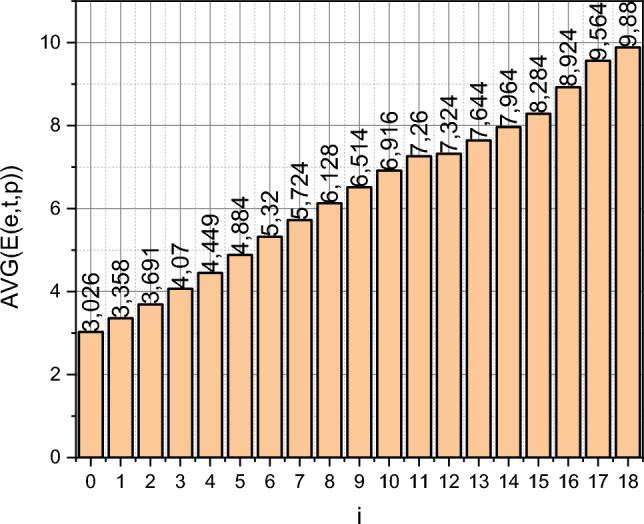
Dataset $$E = f\left( i \right)$$.

Therefore, the expediency of applying the theoretical results of this research in SIEM is obvious. However, such an applied orientation becomes problematic when it is necessary to find an open dataset for testing the proposed system. These circumstances force us to resort to simulation modelling, the data for which is a generalization of open information about the spread of the Petya encryption virus. On June 27, 2017, victims of this virus were Ukrainian companies and Ukrainian branches of international companies, including Nova Poshta, Zaporizhzhiaoblenergo, Dniproenergo, Oshchadbank, media holding Lux, Mondelēz International, TESA, Nivea, Mars, mobile operators LifeCell, UkrTeleCom, Kyivstar and many others. In Kyiv, in particular, some ATMs and cash terminals in stores were found to be infected. It was in Ukraine that the first attacks were recorded. The authors summarized the information available on the IT community website https://habr.com regarding the spread of the Petya virus in the form of a dataset visualized in Fig. 1. The ordinate axis is graduated in thousand c.u. and represents the average number of infected computing devices $E$. The abscissa axis is graduated in time intervals $\Delta$. For further research, we will select the training, test and forecast samples within the available data: $T_{e} \in i = \left[ {0,6} \right]$, $T_{t} \in i = \left[ {7,11} \right]$, $T_{tr} = T_{e} \cup T_{t}$, $T_{p} \in \left[ {12,18} \right]$. Since variability is characteristic of the $E = f\left( i \right)$ measurements, we further take it into account by defining a stochastic vector $\varepsilon$ with independent stochastic interval elements $\varepsilon \left( i{\Delta } \right) \in \left[ {\varepsilon_{ - } ,\varepsilon_{ + } } \right]$, $i = \overline{0,18}$.

We will carry out further calculations by applying the following three sets of intervals for the generation coefficient $b$ and the death coefficient $m$:

$$\begin{aligned} I_{i} & = \left\{ {i = \overline{{\left\{ {1,3} \right\}}} :\left( {I_{b}^{\left( i \right)} ,I_{m}^{\left( i \right)} } \right)} \right\} \\ & = \left( {1:\left( {0,075;0,100} \right),\left( {0,050;0,065} \right);} \right. \\ & \quad 2:\left( {0,075;0,125} \right),\left( {0,050;0,090} \right); \\ & \quad \left. {3:\left( {0,055;0,065} \right),\left( {0,035;0,045} \right)} \right). \\ \end{aligned}$$

The interval for the limits of variation of the model's output parameter is set as $${\rm E} = E_{j} = \left[ { - 0,5;0,5} \right]$$$$\forall j \in \left[ {0,N_{e} } \right]$$, $$N_{e} = 6$$. Let's apply the mathematical apparatus presented in “Models and methods” for the analysis of the output data on the training, test and forecast intervals $$\left\langle {T_{e} ,T_{t} ,T_{p} } \right\rangle$$, respectively.

**Figure 2:**
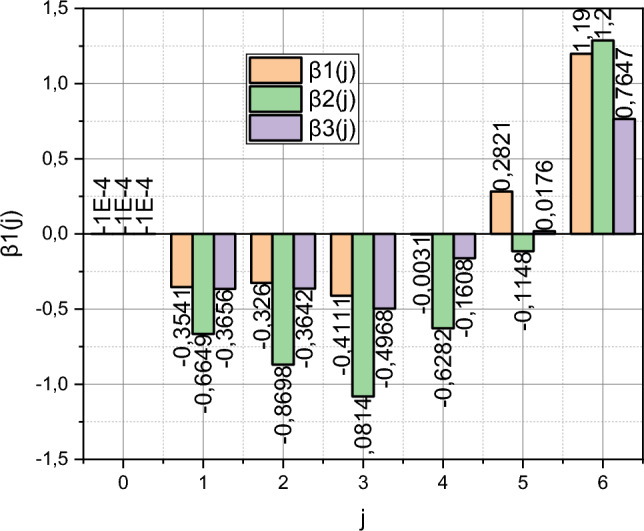
Visualization of the dependency $\left\{ {\beta_{j}^{\left( i \right)} } \right\} = f\left( {\left\{ {I_{b}^{\left( i \right)} ,I_{m}^{\left( i \right)} } \right\}} \right)$, $i = \overline{1,3}$, $j = \overline{0,6} \in T_{e}$.

The training interval summarizes the data $T_{e} \in i = \left[ {0,6} \right]$. The residual function (30) contains two integral components that can only be evaluated numerically. For this, a combination of several quadrature formulas, generalized by the Tiled method, implemented in the Matlab engineering software package as a *quad2d* function, was used. The essence of this method is to divide the area of integration into a set of trapezoidal or rectangular areas. This choice is justified by the fact that the Trust Region method represented in Matlab by the *lsqnonlin* function was then used to minimize the discrepancy $J\left( \beta \right)$. Function *lsqnonlin* optimized for use with a function of the quadratic norm type. The use of the *lsqnonlin* function $J\left( \beta \right) = 10^{ - 3}$ made it possible to calculate the value of the Lagrange multipliers ${\rm B} = \left\{ {\beta_{j}^{\left( i \right)} } \right\} = f\left( {\left\{ {I_{b}^{\left( i \right)} ,I_{m}^{\left( i \right)} } \right\}} \right)$, $i = \overline{1,3}$, $j = \overline{0,6} \in T_{e}$. The results of the calculations are presented in Fig. 2.

**Figure 3:**
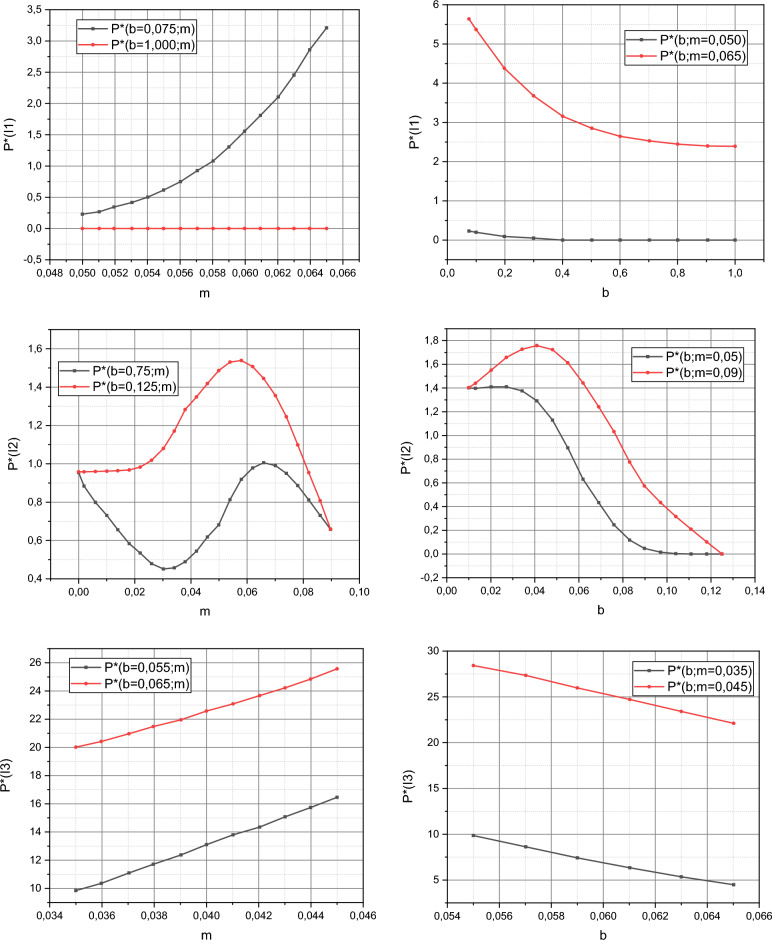
Visualization of the dependency $P^{ * } = f\left( {I_{i} ,b,m} \right)$, $i = \overline{1,3}$.

**Figure 4:**
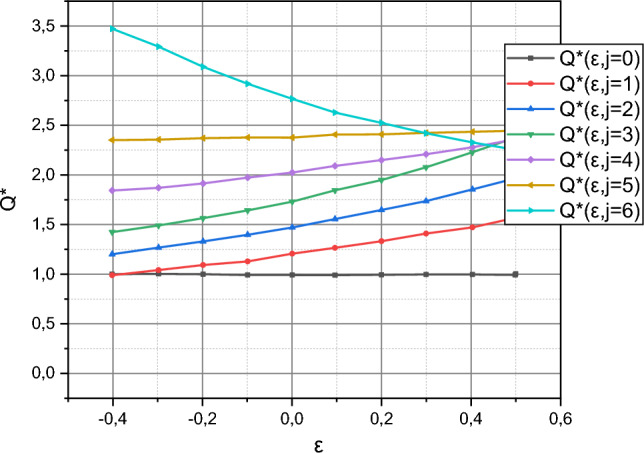
Visualization of the dependency $Q^{ * } = f\left( {\varepsilon ,j} \right)$, $j = \overline{{0,N_{e} }}$, $N_{e} = 6$.

The known values of the Lagrange multipliers ${\rm B}$ make it possible to implement the reverse course and calculate the values of the functions $P^{ * } = f\left( {I_{i} ,b,m} \right)$, $i = \overline{1,3}$, and $Q^{ * } = f\left( {\varepsilon ,j} \right)$, $j = \overline{{0,N_{e} }}$, $N_{e} = 6$, using expressions (25) and (26), respectively. The calculated dependencies are presented in Figs. 3 and 4. Note that the three-dimensional dependence $P^{ * } = f\left( {I_{i} ,b,m} \right)$ for ease of perception is presented in 2D projections for the limit values of the characteristic parameters $\left\langle {b,m} \right\rangle$. Boundary values are the limits of intervals for these variables, summarized by sets $I_{i}$, $i = \overline{1,3}$.

After training the model (18), we will proceed to its testing. The test interval summarizes the data $T_{t} \in i = \left[ {7,11} \right]$ (see Fig. 1). The output parameter $E^{\left( t \right)}$ is calculated according to the expression (23), where $b$ and $m$ are stochastic parameters with a compatible function of the probability distribution density $P^{ * } \left( {b,m} \right)$ and $\varepsilon \left(i {\Delta } \right)$ is the stochastic coefficient of variability of the measurements of the output parameter of the object with the functions of the probability distribution density $q_{i} \left( {\varepsilon \left( {i\Delta } \right)} \right) \in Q^{ * }$, $i \in \left[ {7,11} \right]$. To generate trajectories of stochastic parameters $b$, $m$, $\varepsilon \left(i {\Delta } \right)$, $i \in \left[ {7,11} \right]$, a 2D adaptation of the Ulam–Neumann exception method^42^ with the volume of the generated sample $k = 10^{5}$ was used. Each exponential trajectory is determined by a pair of values of stochastic parameters $b$, $m$ and the value of the stochastic parameter $\varepsilon \left( i{\Delta } \right)$ is added to the value of each $i \Delta$-th point of this trajectory by its probability distribution density. The resulting trajectory can no longer be classified as exponential. The only deterministic parameter that affects the set of trajectories is the number of infected computing devices at the initial moment $i = 0$.

**Figure 5:**
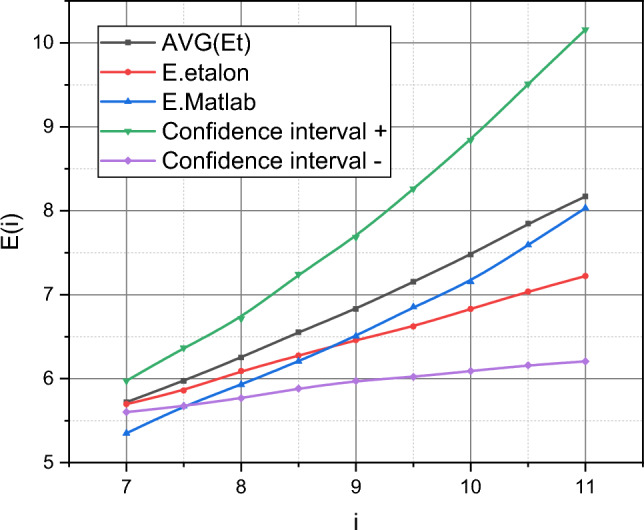
Visualization of a family of curves $E = f\left( i \right)$, $i \in \left[ {7,11} \right] = T_{t}$.

The test results are presented in Fig. 5 by the family of curves $E = f\left( i \right)$, $i \in \left[ {7,11} \right] = T_{t}$. The curve $\overline{E}^{\left( t \right)} = f\left( i \right)$ is the averaged trajectory as a result of the description of the interval $T_{t}$ by the trained model (18) at the limit values of the stochastic parameters $b$ and $m$ imposed by the set $I_{2}$.

The curve $E_{etalon} = f\left( i \right)$ is a visualization of the values of the function $E\left( i \right)$, $i = \overline{7,11}$, from Fig. 1. The curve $E_{Matlab} = f\left( i \right)$ demonstrates the result of describing the dependence $E\left( i \right)$, $i = \overline{7,11}$, on the technological capabilities of standard Matlab functions in the manner described on the page https://uk.mathworks.com/help/ident/ug/forecasting-predator-prey-populations.html for the initial data $E\left( i \right)$, $i = \overline{0,10}$, from Fig. 1. Finally, the curves $\left\{ {E_{ +CI } ,E_{ CI- } } \right\} = f\left( i \right)$ represent the limits of the confidence interval of the variance of the values of $E^{\left( t \right)} = f\left( i \right)$, $i \in T_{t}$, obtained using the trained model (18).

The absolute characteristics of the quality of testing are the root mean square deviation $\delta$ and the relative deviation $\xi$ of the empirical data from the etalon data. We will give analytical expressions adapted to the conditions of the experiment for calculating these characteristics:

$$\begin{aligned} \delta = & \sqrt {\sum\limits_{i = 7}^{11} {\left( {E\left( {i\Delta } \right) - E^{\left( t \right)} \left( {i\Delta } \right)} \right)^{2} } } , \\ \xi = & {\delta \mathord{\left/ {\vphantom {\delta {\sqrt {\sum\limits_{i = 7}^{11} {\left( {E\left( {i\Delta } \right)} \right)^{2} } + } }}} \right. \kern-0pt} {\sqrt {\sum\limits_{i = 7}^{11} {\left( {E\left( {i\Delta } \right)} \right)^{2} } + } }}\sqrt {\sum\limits_{i = 7}^{11} {\left( {E^{\left( t \right)} \left( {i\Delta } \right)} \right)^{2} } } , \\ \end{aligned}$$

where the values $E\left( {i\Delta } \right)$ are taken from Fig. 1, and the values $E^{\left( t \right)} \left( {i\Delta } \right)$ are generated by the trained model (18). As a result of the generalization of those shown in Fig. 4 empirical results in metric, $\left\langle {\delta ,\xi } \right\rangle$ we got:

$$\left\langle {\delta^{\left( t \right)} ,\xi^{\left( t \right)} } \right\rangle = \left( {1,2647;0,0417} \right),\quad \left\langle {\delta_{Matlab} ,\xi_{Matlab} } \right\rangle = \left( {0,9520;0,0321} \right).$$

**Figure 6:**
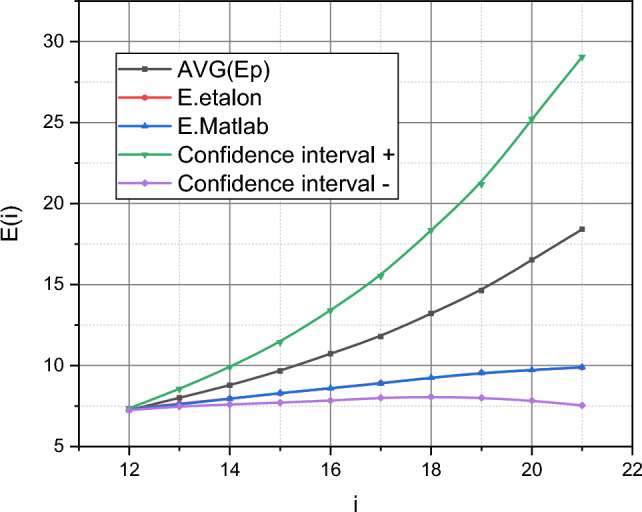
Visualization of a family of curves.

Finally, we conclude “Results” by applying the trained model (18) directly to forecasting. It should describe the value of the function $E = f\left( i \right)$ from Fig. 1, $i \in \left[ {12,18} \right] = T_{t}$. Moreover, $i = \left[ {16,18} \right]$ $\Delta : = 2\Delta$, i.e., these measurements of the output data were carried out with a twice-extended interval. It is this fact that explains the extended range of changes $i$ in Fig. 6 to $i = 21$. The etalon data is for counts $i = \left\{ {12 \div 15,17,19,21} \right\}$.

The forecasting results are presented in Fig. 6 by the family of curves $E = f\left( i \right)$, $i \in \left[ {12,21} \right] = T_p$. The curve $\overline{E}^{\left(p \right)} = f\left( i \right)$ is the averaged trajectory as a result of the description of the interval $T_p$ by the model (18) trained on the interval $T_p$ at the limit values of the stochastic parameters $b$ and $m$ imposed by the set $I_{2}$.

The curve $E_{etalon} = f\left( i \right)$ is a visualization of the values of the function $E\left( i \right)$, $i = \left\{ {12 \div 15,17,19,21} \right\}$, from Fig. 1. The curve $E_{Matlab} = f\left( i \right)$ demonstrates the result of describing the dependence $E\left( i \right)$, $i \in \left[ {12,21} \right]$, on the technological capabilities of standard Matlab functions in the manner described on the page https://uk.mathworks.com/help/ident/ug/forecasting-predator-prey-populations.html for the initial data $E\left( i \right)$, $i = \overline{0,10}$, from Fig. 1. The curves $\left\{ {E_{CI + } ,E_{CI - } } \right\} = f\left( i \right)$ represent the limits of the confidence interval of the variance of the values $E^{\left(p \right)} = f\left( i \right)$, $i \in T_p$, obtained using the trained model (18).

## Discussion

The last decade can without exaggeration be called the "decade of neural networks". Bold experiments with architectures of deep neural networks and their ensembles in combination with the use of Big Data for training allowed us to achieve truly impressive results in solving such classical problems of pattern recognition theory as classification and identification. But have neural networks become smarter? Let's recall the classic flaw of neural networks—overfitting. The essence of this problem is that the neural network model, perceiving only instances from the training sample, adapts to them instead of learning to classify them. Simply put, overfitting is when a neural network in the training process "remembers" the training sample instead of "generalizing" it. In principle, with an infinitely large training sample, the problem of overfitting disappears. But when we talk about the so-called "small data" this postulate does not work. It is when analyzing small data that the problem of overfitting manifests itself in full. When analyzing small data for their classification and identification, one should resort to the methods of machine learning, and not artificial intelligence. This is exactly what the authors did in the context of the task of forecasting the development of a cyber epidemic at an early stage.

Let's take a closer look at the training data, represented in the form of a diagram in Fig. 1. Data visualization instead of a tabular form of their presentation was not chosen by the authors by chance. Figure 1 demonstrates the dynamics of the development of the cyber epidemic of the spread of the Petya encryption virus as it was presented to the general public. We see, in fact, the linear dynamics of the development of this process. Frankly, this immediately raised suspicions among the authors, because intuitively it seems that such a process should develop exponentially until the "cavalry from over the hill" appears in the form of a specialized defence mechanism, which will mark the break of the exponential. But if we start from direct data, then we see linear dynamics. This is exactly what the standard methods of forecasting numerical series, presented in Matlab, "saw" (see curves $E_{Matlab} = f\left( i \right)$ in Figs. 5 and 6). And if the volume of the test sample was too small for them, which was reflected in the inaccurate determination of the angle of inclination of the line $E_{Matlab} = f\left( i \right)$ relative to the line $E_{etalon} = f\left( i \right)$ in Fig. 5, then on Fig. 6, these lines practically coincided.

Now let's look at the functions $\overline{E}^{\left( t \right)} = f\left( i \right)$ and $\overline{E}^{\left(p \right)} = f\left( i \right)$ presented in Figs. 5 and 6, respectively. The function $\overline{E}^{\left( t \right)} = f\left( i \right)$ is also linear, which represents the analytical flexibility embedded in the mathematical model (18). At the same time, the values of the function $\overline{E}^{\left( t \right)} = f\left( i \right)$ stably prevail over the values of the function $E_{etalon} = f\left( i \right)$, $i = \overline{7,11}$. That is, the model (18) trained on the data of interval $T_e$ "prepares for the worst". Finally, the difference will appear in Fig. 6. The function $\overline{E}^{\left(p \right)} = f\left( i \right)$ shows an increasing nonlinear character. How can such results be explained? There are two explanations. Or the trained model (18) is inadequate for forecasting the data represented in Fig. 1, or these initial data are incomplete or intentionally distorted.

The authors can reasonably reject the first option. To do this, we recall that the stochastic characteristic parameters $b$, $m$, $\varepsilon \left( {i\Delta } \right)$ take values from the intervals, the limit ranges of which are embodied in the set of sets $I_{i}$, $i = \overline{1,3}$. Recall that the curves $\overline{E}^{\left( t \right)} = f\left( i \right)$ and $\overline{E}^{\left( p\right)} = f\left( i \right)$ were obtained under the condition that the values of the parameters $b$, $m$, $\varepsilon \left( i{\Delta } \right)$ satisfy the set $I_{2}$. Now recall that $b$ and $m$ are stochastic parameters with a compatible function of the probability distribution density $P^{ * } \left( {b,m} \right)$, and $\varepsilon \left(i {\Delta } \right)$ is a stochastic coefficient of variability of measurements of the output parameter of the object with functions of the probability distribution densities $q_{i} \left( {\varepsilon \left( {i\Delta } \right)} \right) \in Q^{ * }$. Let's pay attention to the dependencies $P^{ * } = f\left( {I_{2} ,b,m} \right)$ shown in Fig. 3. Non-linearity is characteristic of this dependence. This is the source of the nonlinearity of the function $\overline{E}^{\left( p\right)} = f\left( i \right)$ shown in Fig. 6. The authors did not accidentally define the set $I_{3}$. Let's pay attention to its characteristics in the form of dependencies $P^{ * } = f\left( {I_{3} ,b,m} \right)$ from Fig. 3, both of which have a linear character. The authors trained the model (18) taking into account that its characteristic parameters satisfied the conditions of the set $I_{3}$. In the qualitative metric $\left\langle {\delta ,\xi } \right\rangle$ the obtained result is characterized by the values $\left\langle {\delta^{\left( t \right)} ,\xi^{\left( t \right)} } \right\rangle_{{I_{3} }} = \left( {0,6676;0,0227} \right)$, i.e. it prevails over the results obtained using standard Matlab methods (recall: $\left\langle {\delta_{Matlab} ,\xi_{Matlab} } \right\rangle = \left( {0,9520;0,0321} \right)$). Thus, the functionality of model (18) for solving the problem of forecasting variable small data using the example of forecasting the development of a cyber epidemic of the spread of the Petya encryption virus can be considered proven. The publicly available data on the development of this cyber epidemic was incomplete and the trained model (18) responded differently from the overfitted standard model from the Matlab environment.

It remains to clarify a few more points regarding the material presented in “Results”. The first point is the definition of the set $I_{1}$. If you look at its characteristics in the form of dependencies $P^{ * } = f\left( {I_{1} ,b,m} \right)$ from Fig. 3, then it becomes obvious that this set is a compromise between the "nonlinear" set $I_{2}$ and the "linear" set $I_{3}$. The authors recommend using the set $I_{1}$ if the initial data is difficult to pre-characterize. The second point is the influence of the stochastic coefficient of variability of measurements $\varepsilon \left(i {\Delta } \right)$ with functions of the probability distribution densities $q_{i} \left( {\varepsilon \left( {i\Delta } \right)} \right) \in Q^{ * }$, $i \in T$, on forecasting results. It is impossible to unambiguously answer this question in numerical and parametric form based on the conducted research. This point needs additional investigation in the context of implementing proactive technologies of AI-powered protection of assets against cyberattacks^46–48^. However, these aspects do not affect the functionality and adequacy of the material presented in the article.

The essence of the author's method is the idea of estimating the model parameter's probability distributions from a small amount of real empirical data, in the representation of which the measurement noise probability distributions are taken into account. The method returns distributions with maximum entropy, which characterize the state of the greatest uncertainty of the studied process. This makes it possible to interpret the resulting forecasts as the most “negative” ones. This circumstance suggests that the author's method may be appropriate for determining pessimistic scenarios when analyzing the reliability of critical systems in conditions of incomplete or distorted telemetry data. This direction can be developed taking into account the fact that the authors previously proposed a mathematical apparatus for describing the influence of complex negative factors on an information system for critical use based on the Markov processes theory^49–51^.

## Conclusions

Security Information and Event Management technologies play an important role in the architecture of modern cyber protection tools. One of the main scenarios for the use of SIEM is the detection of attacks on protected information infrastructure. Consorting that ISO 27001, NIST SP 800-61, and NIST SP 800-83 standards objectively do not keep up with the evolution of cyber threats, research aimed at forecasting the development of cyber epidemics is relevant.

The article proposes a stochastic concept of describing variable small data on the Shannon entropy basis. The core of the concept is the description of small data by linear differential equations with stochastic characteristic parameters. The practical value of the proposed concept is embodied in the method of forecasting the development of a cyber epidemic at an early stage (in conditions of a lack of empirical information). In the context of the research object, the stochastic characteristic parameters of the model are the generation rate, the death rate, and the independent coefficient of variability of the measurement of the initial parameter of the research object. Analytical expressions for estimating the probability distribution densities of these characteristic parameters are proposed. It is assumed that these stochastic parameters of the model are imposed on the intervals, which allows for manipulation of the nature and type of the corresponding functions of the probability distribution densities. The task of finding optimal functions of the probability distribution densities of the characteristic parameters of the model with maximum entropy is formulated. The proposed method allows for generating sets of trajectories of values of characteristic parameters with optimal functions of the probability distribution densities. The example demonstrates both the flexibility and reliability of the proposed concept and method in comparison with the concepts of forecasting numerical series implemented in the base of Matlab functions.

The authors see the direction of *further research* in deepening the understanding of the influence of the variability of measurements of the output parameter of the research object on the results of evaluation and forecasting of small data. This direction could be added by enhancing protection means against AI-powered attacks^52,53^.

## Data Availability

Most data is contained within the article. All the data is available on request due to restrictions e.g. privacy or ethics. Prof. Viacheslav Kovtun (kovtun_v_v@vntu.edu.ua) will be glad to answer any questions regarding the data mentioned in the article.
